# Real-World Outcomes of Crizotinib in ROS1-Rearranged Advanced Non-Small-Cell Lung Cancer

**DOI:** 10.3390/cancers16030528

**Published:** 2024-01-26

**Authors:** Hyeon Hwa Kim, Jae Cheol Lee, In-Jae Oh, Eun Young Kim, Seong Hoon Yoon, Shin Yup Lee, Min Ki Lee, Jeong Eun Lee, Chan Kwon Park, Kye Young Lee, Sung Yong Lee, Seung Joon Kim, Jun Hyeok Lim, Chang-min Choi

**Affiliations:** 1 Division of Pulmonology and Critical Care Medicine, Department of Internal Medicine, Asan Medical Center, University of Ulsan College of Medicine, 88 Olympic-ro 43-gil, Songpa-gu, Seoul 05505, Republic of Korea; hyeonhwa1@gmail.com; 2 Department of Oncology, Asan Medical Centre, University of Ulsan College of Medicine, Seoul 05505, Republic of Korea; jclee@amc.seoul.kr; 3 Department of Internal Medicine, Chonnam National University Medical School and Hwasun Hospital, Gwangju 58128, Republic of Korea; droij@chonnam.ac.kr; 4 Division of Pulmonary and Critical Care Medicine, Department of Internal Medicine, Severance Hospital, Yonsei University College of Medicine, Seoul 03722, Republic of Korea; 5 Department of Internal Medicine, Pusan National University Yangsan Hospital, Yangsan 50612, Republic of Korea; dognose79@naver.com; 6 Department of Internal Medicine, Kyungpook National University School of Medicine, Daegu 41404, Republic of Korea; 7 Division of Pulmonology, Allergy and Critical Care Medicine, Department of Internal Medicine, Pusan National University Hospital, Busan 49241, Republic of Korea; leemk@pusan.ac.kr; 8 Division of Pulmonology, Department of Internal Medicine, Chungnam National University, Daejeon 34134, Republic of Korea; 9 Division of Pulmonary, Allergy and Critical Care Medicine, Department of Internal Medicine, Yeouido St. Mary’s Hospital, College of Medicine, The Catholic University of Korea, Seoul 16247, Republic of Korea; ckpaul@catholic.ac.kr; 10 Departments of Internal Medicine, Konkuk University Medical Center, Konkuk University School of Medicine, Seoul 05030, Republic of Korea; kyleemd@kuh.ac.kr; 11 Division of Pulmonary, Allergy, and Critical Care Medicine, Department of Internal Medicine, Korea University Guro Hospital, Korea University College of Medicine, Seoul 08308, Republic of Korea; 12 Division of Pulmonology, Department of Internal Medicine, College of Medicine, The Catholic University of Korea, Seoul 16247, Republic of Korea; cmcksj@catholic.ac.kr; 13 Department of Internal Medicine, Inha University Hospital, Incheon 22332, Republic of Korea

**Keywords:** ROS1, non-small-cell lung cancer, crizotinib, real-world efficacy

## Abstract

**Simple Summary:**

Crizotinib, an oral tyrosine kinase inhibitor that targets ALK, MET, and ROS1 kinase, has emerged as an effective treatment for ROS1-rearranged NSCLC. In South Korea’s real-world setting, characterized by a higher elderly population and increased brain/central nervous system metastasis rates, crizotinib remains a cornerstone in treating ROS1-rearranged NSCLC, providing lasting clinical benefits with a favorable safety profile. Liquid biopsies, utilizing biomarkers, are instrumental in detecting targetable mutations, monitoring the disease burden, and identifying resistance mechanisms. In our study, next-generation sequencing using cell-free total nucleic acids enables the detection of ROS1 fusions and the identification of resistance mechanisms during disease progression.

**Abstract:**

Real-world data on the use and outcomes of crizotinib in ROS1-rearranged non-small-cell lung cancer (NSCLC) are limited. This study aims to analyze the real-world efficacy of crizotinib in South Korea and explore the utilization of liquid biopsies that implement next-generation sequencing (NGS) using cell-free total nucleic acids. In this prospective multicenter cohort study, 40 patients with ROS1-rearranged NSCLC, either starting or already on crizotinib, were enrolled. Patients had a median age of 61 years, with 32.5% presenting brain/central nervous system (CNS) metastases at treatment initiation. At the data cutoff, 48.0% were still in treatment; four continued with it even after disease progression due to the clinical benefits. The objective response rate was 70.0%, with a median duration of response of 27.8 months. The median progression-free survival was 24.1 months, while the median overall survival was not reached. Adverse events occurred in 90.0% of patients, primarily with elevated transaminases, yet these were mostly manageable. The NGS assay detected a CD74–ROS1 fusion in 2 of the 14 patients at treatment initiation and identified emerging mutations, such as ROS1 G2032R, ROS1 D2033N, and KRAS G12D, during disease progression. These findings confirm crizotinib’s sustained clinical efficacy and safety in a real-world context, which was characterized by a higher elderly population and higher rates of brain/CNS metastases. The study highlights the clinical relevance of liquid biopsy for detecting resistance mechanisms, suggesting its value in personalized treatment strategies.

## 1. Introduction

Lung cancer remains a predominant health concern worldwide, including in South Korea, due to its high prevalence and mortality rates [1,2]. In 2021, South Korea reported approximately 18,900 lung-cancer-related deaths, accounting for 22.9% of all cancer-related mortality [3]. Notably, non-small-cell lung cancer (NSCLC) accounts for approximately 80% of these cases, and nearly half of patients were diagnosed at stage IV of the disease with poor 5-year survival rates of <10% [2,4,5]. For patients with metastatic NSCLC, platinum-based chemotherapy has historically been the mainstay treatment, albeit with limited response rates of 15–30% and only modest improvements in survival [6,7,8].

However, the identification of specific gene mutations and the subsequent introduction of targeted therapies has dramatically changed the landscape of NSCLC treatment [9,10,11]. In particular, crucial biomarkers for cancer detection and treatment efficacy in NSCLC have been identified in key genetic mutations: Epidermal growth factor receptor (EGFR), anaplastic lymphoma kinase (ALK), V-Raf murine sarcoma viral oncogene homolog B1 (BRAF), Kirsten rat sarcoma viral oncogene homolog (KRAS), and the c-ros oncogene 1 (ROS1) [12,13,14,15]. The treatments targeting these genetic mutations include tyrosine kinase inhibitors (TKIs), monoclonal antibodies, and antibody–drug conjugates, which have demonstrated higher efficacy and lower toxicity than traditional therapies [12,16]. Among the targetable mutations, ROS1 rearrangement is an actionable driver oncogene, which is prevalent in approximately 1–2% of NSCLC patients globally and 2–3% among the East Asian populations [17,18,19,20]. It is distinctive for its prevalence among younger populations, a higher proportion of women, and a larger percentage of never-smokers, paralleling ALK-rearranged NSCLC [20,21].

Crizotinib is an oral tyrosine kinase inhibitor (TKI) that targets ALK, mesenchymal epithelial transition factor receptor (MET), and ROS1 tyrosine kinase, and it has become a potent treatment for ROS1-rearranged NSCLC [22,23]. In March 2016, the Food and Drug Administration approved crizotinib as the first targeted therapy for ROS1-rearranged NSCLC, which remains a pivotal cornerstone. Crizotinib has consistently shown high response rates and high median progression-free survival (PFS). In the phase I PROFILE 1001 trial, a standard dosage of 250 mg twice daily resulted in an objective response rate (ORR) of 72% and a median PFS of 19.2 months [24]. Similarly, a phase II trial in an East Asian population reported an ORR of 69.3% and a median PFS of 13.4 months [25]. While these results from randomized clinical trials (RCTs) are promising, translating this evidence to real-world clinical settings presents challenges. Patients in daily practice often have a range of comorbidities and characteristics not typically represented in highly controlled RCT environments, leading to potential gaps between the trial efficacy and real-world effectiveness [26,27]. Early real-world studies yielded consistent outcomes, with ORRs ranging from 80 to 87% and the median PFS ranging from 9.1 to 18.4 months, indicating its potential effectiveness in routine clinical practice [28,29].

Advancements in lung cancer treatments have heightened the demand for innovative diagnostic and treatment strategies, particularly in molecular diagnostics. Liquid biopsies, a minimally invasive approach, are a key technology in this area [30,31]. This technology offers crucial insights into specific biomarkers for targeted therapies by analyzing body fluids such as blood, urine, sputum, and saliva. These fluids can reveal a range of biomarkers, including cell-free DNA (cfDNA), circulating tumor DNA, cell-free RNA (cfRNA), circulating tumor cells, exosomes, and DNA-methylated fragments. Thus, liquid biopsies have shown promise in providing insights into diagnosis, treatment targets, and resistance mechanisms [31,32,33]. The potential of a liquid biopsy extends to ROS1-rearranged NSCLC; however, the data from previous studies on ROS1-rearranged NSCLC are limited compared to other molecular subsets of NSCLC [34,35].

Despite the available data on crizotinib for ROS1-rearranged NSCLC patients from clinical studies, real-world information on its use and outcomes beyond these strict trial settings remains limited [25,36,37,38], and hence the efficacy of crizotinib in ROS1-rearranged lung cancer patients with brain metastasis is also yet to be evaluated. Moreover, reliable biomarkers to assess the efficacy of crizotinib are still to be determined. In this study, we aimed to comprehensively analyze the clinical characteristics of patients treated with crizotinib and assess its real-world efficacy through the development of a patient cohort within the regular clinical practice environment in South Korea. Additionally, we explored the clinical relevance of utilizing a liquid biopsy implementing amplicon-based next-generation sequencing (NGS) using cell-free total nucleic acids (cfTNA, cfDNA, and cfRNA) in ROS1-rearranged NSCLC patients.

## 2. Methods

### 2.1. Study Design and Patient Eligibility

This prospective multicenter cohort study enrolled patients with ROS-1 rearranged NSCLC who were starting or already on crizotinib between August 2020 and June 2021. The eligibility criteria were as follows: (1) age of 19 years or above, (2) initial diagnosis of locally advanced or metastatic NSCLC, (3) histological or cytological confirmation of adenocarcinoma with ROS1 rearrangement, and (4) currently taking or about to start crizotinib as a first-line or subsequent therapy. The exclusion criteria were other active or previous malignancies, the completion of crizotinib treatment, and being unsuitable for medical record review. Data were analyzed up to 5 September 2023.

The presence of ROS1 rearrangement was confirmed using immunohistochemistry (IHC), fluorescence in situ hybridization (FISH), or reverse transcription quantitative polymerase chain reaction (RT-qPCR). The presence of EGFR mutations or ALK rearrangements was evaluated using IHC or FISH. The disease evaluation was based on the Response Evaluation Criteria in Solid Tumors (RECIST) version 1.1. Each medical center’s Institutional Review Board approved the study protocol, and informed consent was obtained from all participants before enrollment.

### 2.2. Treatment

Patients were administered 250 mg crizotinib twice daily, and the doses were adjusted for adverse events (AEs) or as deemed necessary by the investigator. The treatment continued until there was evidence of disease progression as defined by the RECIST version 1.1 criteria, unacceptable toxicity, or withdrawal of consent. However, patients could continue the treatment if they still benefited clinically after disease progression. Patients did not receive any other chemotherapy concurrently with crizotinib.

### 2.3. Study Endpoints

The primary efficacy endpoint was the ORR, defined as the proportion of patients who achieved the best response to crizotinib of either a partial response (PR) or a complete response (CR), as determined by conducting a radiological review of each center. The secondary efficacy endpoints included the duration of response (DR), disease control rate (DCR), PFS, and overall survival (OS). DR was defined as the time from the first objective tumor response (CR or PR) to its progression or any-cause death. DCR was defined as the percentage of patients who achieved CR, PR, or stable disease. For a more detailed analysis, PFS was categorized as PFS1 (the duration from the initiation of crizotinib treatment to the initial noted objective tumor progression or death during the treatment, whichever came first) or PFS2 (the same initial period but in the case of the continuation of crizotinib, extended to the subsequent tumor progression prompting crizotinib discontinuation or death, whichever occurred first). OS was defined as the time from the start of the first chemotherapeutic agent to any-cause death. Safety and tolerability were assessed by grading AEs using the Common Terminology Criteria for Adverse Events (version 4.0) and monitoring the dose adjustments.

### 2.4. Sample Preparation and Sequencing Analysis

cfTNA was extracted from frozen plasma using the MagMAX Cell-Free Total Nucleic Acid Isolation Kit and quantified using the Qubit dsDNA high-sensitivity kit (Thermo Fisher Scientific, Waltham, MA, USA). From 15 μL of the eluted cfTNA, 10 μL (the maximum volume of input recommended by the manufacturer) was used for the library preparation if the total amount of cfTNA did not exceed 20 ng. The library preparation was performed using the Oncomine Pan-Cancer Cell-Free Assay (an off-the-shelf panel that targets 272 amplicons within 52 known cancer genes), and sequencing was conducted using the Ion Chef System and Ion S5 XL System (Thermo Fisher Scientific). The data analysis was performed using Torrent Suite version 5.16.1 (Thermo Fisher Scientific) and Ion Reporter Software 5.20.2.0 (Thermo Fisher Scientific), including sequence alignment with the hg19 human reference genome and variant calling.

### 2.5. Statistical Analysis

The analyses of efficacy and safety included all patients who received at least one dose of crizotinib. Continuous variables are presented as means with standard deviation, with categorical variables as percentages. Confidence intervals (CIs) were calculated for all endpoint analyses, if applicable. The Kaplan–Meier method was conducted to analyze the time-to-event data (DR, OS, and PFS) to estimate the median event times using two-sided 95% CIs. All analyses were conducted using IBM SPSS Statistics, version 24.0 (IBM Corporation, Armonk, NY, USA) and SAS statistical software, version 9.4 (SAS Institute, Inc., Cary, NC, USA).

## 3. Results

### 3.1. Patient Characteristics

The eligibility screening yielded 40 patients who started crizotinib for ROS1-rearranged adenocarcinoma between November 2018 and June 2021. Of them, 25 (62.5%) patients had initiated crizotinib before enrollment, and 15 (37.5%) patients initiated crizotinib at the time of enrollment. The median follow-up duration from crizotinib initiation to the end of the study or death was 29.5 months (interquartile range (IQR), 24.1–36.6). The demographic and clinical characteristics at study entry are detailed in Table 1. The median age was 60.5 years (IQR, 27–83), and 26 (65.0%) were female. Based on the available data (*n* = 39), 27 patients (69.2%) were never-smokers. The European Cooperative Oncology Group (ECOG) performance statuses were as follows: 0 in 17.5%, 1 in 75.0%, and 2 in 7.5% of cases. At the time of the initial diagnosis, 32 (80.0%) were diagnosed with stage IV disease, and the most common metastatic sites were the brain/central nervous system (CNS) and lungs, both at 32.5%. A total of 20 patients (50%) had undergone prior chemotherapy, whereas the other 20 patients were treatment-naïve. The median durations from initial diagnosis to crizotinib initiation were 17 days (IQR, 14–28 days) for patients receiving first-line crizotinib and 14.2 months (IQR, 7.7–46.3 months) for patients with a chemotherapeutic treatment history. A total of 18 underwent pre-crizotinib radiotherapy (lung lesions, *n* = 8; bone lesions, *n* = 5; brain lesions, *n* = 5). ROS1 rearrangement was detected using RT-qPCR (87.5%), IHC (20%), and/or FISH (7.5%). Most patients were tested for EGFR mutations (*n* = 39) and ALK rearrangements (*n* = 37). Among these, none had EGFR mutations or ALK rearrangements [9].

### 3.2. Efficacy of Crizotinib

**Figure 1:**
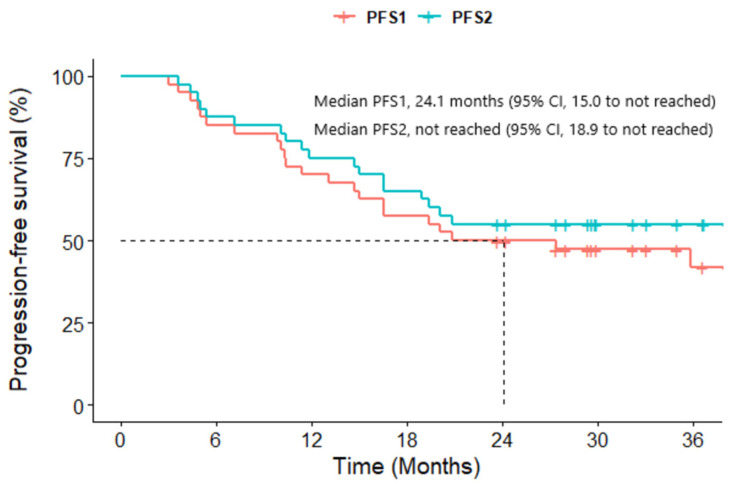
Kaplan–Meier curves of the progression-free survival of 40 ROS1-rearranged NSCLC patients treated with crizotinib. PFS1, denoting the duration from the onset of crizotinib administration to the first observed tumor progression. PFS2, representing the timeframe from starting crizotinib to the progression that led to the termination of crizotinib, even beyond initial progression.

**Figure 2:**
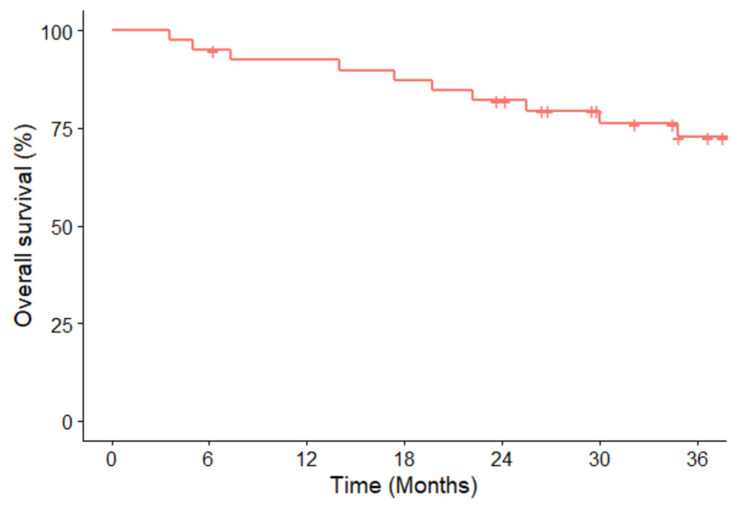
Kaplan–Meier curves of the overall survival of 40 ROS1-rearranged NSCLC patients treated with crizotinib.

The ORR was 70.0% (95% CI, 53.5–83.4). A total of 28 patients achieved a PR, though none achieved a CR. The DCR was recorded at 100% (95% CI, 91.2–100). The median DR was 27.8 months (95% CI, 21.0–34.6) (Table 2). By the data cutoff date, 22 (55.0%) either showed PD (47.5%, *n* = 19) or had died (25.0%, *n* = 10). The location of progression was an extracranial lesion for 11 (27.5%) patients and an intracranial lesion for 8 (20.0%) patients. Of the 19 patients showing PD, 6 (31.6%) remained on crizotinib, perceiving ongoing clinical benefits despite the progression. Subsequently, two of them discontinued crizotinib due to further progression. The median PFS1 was 24.1 months (95% CI, 15.0 to not reached), and the PFS1 rates at 6, 12, and 18 months were 85.0%, 70.0%, and 57.5%, respectively. The median PFS2 was not met by the data cutoff; the PFS2 rates at 6, 12, and 18 months were 85.0%, 72.5%, and 62.5%, respectively (Figure 1). The median OS was also not reached by the data cutoff, with survival probabilities at 6, 12, and 18 months of 95.0%, 92.4%, and 87.3%, respectively (Figure 2).

Brain/CNS metastasis at the time of crizotinib initiation was observed in 13 (32.5%) patients, and 6 (46.2%) underwent other treatments for the brain/CNS during crizotinib treatment: gamma knife radiosurgery, cyberknife, or whole brain radiation therapy. Of the 13 patients, 4 showed only intracranial progression despite all receiving concomitant radiotherapy. Patients with brain metastasis (*n* = 13) showed a marginally significant difference in the median PFS1 compared to patients without brain metastasis (*n* = 27) (13.1 months vs. 35.8 months, *p* = 0.09) (Figure S1).

### 3.3. Safety of Crizotinib

At the data cutoff, the median duration of crizotinib treatment was 22.3 months (IQR, 11.2–35.4), with 19 patients (47.5%) still undergoing the treatment. Permanent treatment discontinuation was due to disease progression (35.0%), AEs (7.5%), and death (5.0%). Approximately 90% of the patients showed AEs of any grade: elevated alanine aminotransferase (37.5%), elevated aspartate aminotransferase (32.5%), nausea (22.5%), and diarrhea (20.0%). All reported AEs were of grade 3 or lower, except for one case of pneumonia (grade 5) (Table 3). Dose adjustments were required for 15 patients (37.5%), and of them, 3 permanently discontinued crizotinib due to AEs (Table S1): pneumonia (*n* = 1), renal abscess (*n* = 1), and sudden hearing loss (*n* = 1).

### 3.4. Amplicon-Based Targeted Sequencing of cfTNA

This study employed cfTNA analysis coupled with NGS to identify the ROS1 rearrangement and resistance mechanisms in a subset of our patient cohort. Among the 14 patients (35.0%) assessed using liquid biopsy at the onset of crizotinib treatment, two exhibited a CD74-ROS1 fusion. In another group of eight patients (20.0%) tested during disease progression, cfTNA analysis with NGS revealed the ROS1 G2032R mutation in one and the ROS1 D2033N mutation in another. Furthermore, a KRAS G12D mutation, EML4–ALK fusion, and KIF5B–RET fusion were each identified in one of these patients. Of the five patients who underwent cfTNA testing both at the start of the treatment and during disease progression, three were negative for ROS1 rearrangement on both occasions, while one patient who initially exhibited the CD74–ROS1 fusion at treatment initiation later demonstrated the ROS1 G2032R mutation upon disease progression (Table S2).

## 4. Discussion

Since its 2016 approval, crizotinib has established itself as the primary treatment for advanced NSCLC patients with ROS1 rearrangement. This prospective multicenter cohort study offers a comprehensive overview of the clinical characteristics and outcomes of 40 patients with ROS1-rearranged adenocarcinoma who received crizotinib in a real-world setting. In this study, we evaluated the clinical significance of using liquid biopsies for ROS1 rearrangement.

The high ORR of 70% (95% CI, 53.5–83.4) observed in our cohort aligns closely with previous studies [25,36,37]. A large phase II study of 127 East Asian patients reported an ORR of 71.7% (63.0–79.3) [25], and a South Korean study reported a similar 73.3% ORR for crizotinib-treated patients [39]. Our patients achieved a DCR of 100%, indicating the significant efficacy of crizotinib. Additionally, the median DR was 27.8 months, demonstrating more durable responses compared to previous studies, which reported a median DR ranging from 19.7 to 24.7 months [25,37]. Recently, real-world studies from Europe and China have reported median PFSs of 9.1 and 18.4 months, respectively, but the median OS has not yet been reached [28,29]. In this study, the median PFS1 exceeded 24 months, and the median OS was also not reached; nevertheless, approximately 90% of patients survived at 12 months. These data compare favorably with other studies [28,36,38], which strengthens the evidence supporting the clinical benefits and durability of crizotinib in treating ROS1-rearranged NSCLC. However, caution is warranted in the interpretation due to our enrollment criteria, which included patients who had initiated crizotinib before enrollment.

In line with previous studies [40,41], our cohort identified ROS1-rearranged NSCLC primarily in female patients (65.0%) and never-smokers (69.2%). However, the median age of our cohort was 60.5 years, which is older than the 51–56-year range reported in other studies [25,28,29]. This suggests that patients receiving crizotinib in real-world settings may be older than those in previous studies, yet still attain high efficacy and durability, irrespective of age. Crizotinib is a less favorable option for patients with brain/CNS metastases due to its limited penetration of the blood–brain barrier. In our real-world study conducted in South Korea, the incidence of brain/CNS metastases at the baseline in ROS1-rearranged NSCLC patients receiving crizotinib was 32.5%, which is higher than in most other studies (3.2–23.1%) [25,28,29,38]. Despite the high incidence of brain/CNS metastases, the ORR was comparable to that reported in other studies [25,28,29,38,42]. Previous studies have demonstrated a consistent ORR of 73.9% for patients with brain metastasis and 71.2% for those without at baseline, though a notable difference in the median PFS was observed (9.4 months vs. 20.0 months) [25,36]. In the present study, patients with brain metastasis had a marginally shorter PFS1 than those without brain metastasis (13.1 months vs. 35.8 months, *p* = 0.09). Among the 13 patients who had initial brain metastases, 4 (30.8%) experienced only intracranial progression despite undergoing concomitant radiation therapy. These findings suggest that while crizotinib does show durable efficacy in patients with brain/CNS metastases, its effectiveness is nevertheless constricted, underscoring the need for newer TKIs with better CNS penetration.

In the present study, 90% of patients experienced AEs of any grade. The safety profile aligned with the findings from previous studies, and elevated transaminase levels and gastrointestinal AEs, such as nausea and vomiting, are reported in both the present study and the literature. However, visual disturbances and bradycardia, which were frequently mentioned in other reports, were less common in our cohort [25,36,37]. We observed a higher rate of dose adjustments (37.5%) than in earlier studies on patients treated with crizotinib (15.7–20.5%) [25,43]. Nonetheless, most AEs became manageable after dose adjustments, and in the present study, only three patients eventually had to discontinue the medication due to AEs. The AEs that led to discontinuation, such as pneumonia, renal abscess, and sudden hearing loss, may not be clearly associated with the treatment. No new safety concerns were further identified in the present study.

In recent studies, the potential of a liquid biopsy based on biomarkers, including cell-free nucleic acids, has garnered attention for diagnostics, prediction, and monitoring treatment response or resistance [31,44,45,46]. NGS, enabling massively parallel sequencing of nucleic acids, facilitates the simultaneous assessment of multiple genes. This has led to the increased use of NGS-based assays in clinical diagnostics for detecting gene rearrangements/fusions. In our study employing amplicon-based NGS with cfTNA, the CD74–ROS1 fusion was detected in 14.3% (2 out of 14) of the patients at the onset of crizotinib treatment. While the limited number of patients that demonstrated the expected results raises the need for cautious interpretation due to the small sample size, it is worth noting that this approach may still hold clinical utility for patients who cannot undergo invasive tissue sampling. For instance, the study by Mezquita et al. [47] reported a detection rate for ROS1 fusion of 67% in treatment-naïve patients, and Dagogo-Jack et al. [48] found a sensitivity of 50% for detecting ROS1 fusion upon relapse after ROS1-directed treatment, both highlighting the challenges in achieving high detection rates. These findings align with previous research indicating lower sensitivity for ROS1 rearrangement detection than for other mutations or oncogenic fusions [49]. To potentially improve the sensitivity of liquid biopsies for detecting ROS1 fusion, enhancements, such as greater probe coverage of the more common fusion partner genes and robust bioinformatics, could be integrated. Additionally, future research is warranted to explore more sensitive methods and increase the size and diversity of the patient cohorts to provide a more representative sampling. Moving beyond mere detection, identifying the resistance mechanisms during disease progression is vital for monitoring the treatment response and assisting clinical decision-making. In recent years, significant efforts have been made to understand the mechanisms of resistance to crizotinib in ROS1-rearranged NSCLC. To date, the most frequently reported ROS1 secondary mutation in patients is the ROS1 G2032R mutation, followed by ROS1 D2033, L2026, and S1986F/Y [50,51,52]. Mutations such as KRAS G12D, BRAF V600E, and KIT D816G have emerged in clinical settings post-crizotinib progression [53,54,55]. Mezquita et al. [47] found ROS1-resistance mutations in 30% (3/10) of patients at progression, and Dagogo-Jack et al. [48] identified genetic alterations mediating resistance in 44% (8/18) upon relapse after ROS1-directed treatment. Consistently, in our cohort, upon disease progression, 25% (2 out of 8) of patients exhibited ROS1 secondary mutations, and 1 (12.5%) patient displayed the KRAS G12D mutation, a known off-target resistance mechanism. Further investigation is needed to understand the potential of other detected mutations, such as the KIF5B–RET and EML4–ALK fusions, as resistance mechanisms and their clinical implications. The early detection of these resistance-mediating mutations using a liquid biopsy could assist in making timely treatment adjustments. Thus, our findings underscore the clinical utility of liquid biopsies in detecting ROS1 fusions and understanding their resistance mechanisms, despite some inherent limitations. Dynamic monitoring through liquid biopsies could be instrumental in shaping personalized treatment strategies, especially with the advent of new TKIs, in the near future.

There were some limitations in our study. Firstly, the sample size was relatively small, reflecting the rarity of ROS1-rearranged NSCLC, which might limit the generalizability of our findings. Secondly, despite the prospective design of the study, the collection of some historical data for patients already undergoing crizotinib treatment at the point of study entry was retrospective, hence the potential biases. Thirdly, due to the small sample size, our findings related to liquid biopsies require validation using studies with larger cohorts. Nevertheless, our study provides valuable insights into the real-world efficacy and safety of crizotinib in patients with ROS1-rearranged NSCLC.

## 5. Conclusions

In conclusion, to the best of our knowledge, the present study is the largest real-world investigation of crizotinib in ROS1-rearranged NSCLC patients in South Korea, therefore highlighting the enduring clinical benefits among Korean patients. In a real-world setting characterized by a higher elderly population and higher brain/CNS metastases rates, crizotinib remains a cornerstone in treating ROS1-rearranged NSCLC. Furthermore, our research highlights the clinical relevance of liquid biopsies as a non-invasive tool for identifying the resistance mechanisms to crizotinib, offering significant implications for personalized treatment approaches.

## Figures and Tables

**Table 1:** Demographic and clinical characteristics of patients receiving crizotinib.

Characteristics	Total (*n* = 40)
Median age, years (range)	60.5 (27–83)
Sex (male/female)	14/26
BMI	24.3 ± 3.2
Smoking status (*n* = 39)	
Never-smoker	27 (69.2)
Former smoker	11 (28.2)
Current smoker	1 (2.6)
ECOG PS	
0	7 (17.5)
1	30 (75.0)
2	3 (7.5)
Stage at initial diagnosis ^a^	
IIA	1 (2.5)
IIB	2 (5.0)
IIIA	3 (7.5)
IIIB	2 (5.0)
IVA	20 (50.0)
IVB	12 (30.0)
Location of biopsy ^b^	
Lung	26 (65.0)
Lymph node	16 (40.0)
Pleural effusion	1 (2.5)
Histologic type	
Adenocarcinoma	40 (100.0)
Metastasis	
Yes	32 (80.0)
No	8 (20.0)
Distant metastatic site at initial diagnosis ^a,b^	
Brain/central nervous system	13 (32.5)
Lung	13 (32.5)
Bone	10 (25.0)
Pleura	9 (22.5)
Adrenal gland	6 (15.0)
Lymph node	6 (15.0)
Liver	2 (5.0)
Other	1 (2.5)
Number of prior chemotherapeutic regimens	
0	20 (50.0)
1	12 (30.0)
2	4 (10.0)
≥3	4 (10.0)
ROS1 rearrangement ^b^	40 (100)
RT-qPCR	38 (95.0)
IHC	8 (20.0)
FISH	3 (7.5)
EGFR mutation (*n* = 39)	0
ALK rearrangement (*n* = 37)	0

Values are presented as means (standard deviation) or numbers (%) unless otherwise indicated. ^a^ “At initial diagnosis” refers more specifically to the time of diagnosis of ROS1-rearranged NSCLC. ^b^ Percentages do not add to 100% as some patients fit multiple categories. ALK; anaplastic lymphoma kinase, BMI; body mass index, ECOG; Eastern Cooperative Oncology Group, EGFR; epidermal growth factor receptor, IHC; immunohistochemistry, RT-qPCR; reverse transcription quantitative polymerase change reaction.

**Table 2:** Summary of efficacy endpoints.

	Total, No. (%)
Number of patients	40
Best overall response	
Complete response	0
Partial response	28 (70.0)
Stable disease	12 (30.0)
ORR (*n* = 40)	28 (70.0)
95% CI	53.5–83.4
DCR (*n* = 40)	40 (100.0)
95% CI	91.2–100
DR	
Median, months (95% CI)	27.8 (21.0–34.6)
PFS1	
Number of events	22 (55.0)
Progressive disease	19 (47.5)
Death without objective progression	3 (7.5)
Median, months (95% CI)	24.1 (15.0–NR)
6 months PFS1 rate, % (95% CI)	85.0 (74.6–96.8)
12 months PFS1 rate, % (95% CI)	70.0 (57.1–85.7)
18 months PFS1 rate, % (95% CI)	57.5 (44.1–75.1)
PFS2	
Number of events	18 (45.0)
Median, months (95% CI)	NR (18.9–NR)
6 months PFS2 rate, % (95% CI)	85.0 (74.6–96.8)
12 months PFS2 rate, % (95% CI)	72.5 (59.9–87.7)
18 months PFS2 rate, % (95% CI)	62.5 (49.2–79.5)
OS	
Number of events	10 (25.0)
Median, months (95% CI)	NR (NR–NR)
6 months OS rate, % (95% CI)	95.0 (88.5–100.0)
12 months OS rate, % (95% CI)	92.4 (84.6–100.0)
18 months OS rate, % (95% CI)	87.3 (77.5–98.4)

CI; confidence interval; DCR, disease control rate; DR, duration of response; ORR, objective response rate; PFS, progression-free survival; NR, not reached.

**Table 3:** Adverse events in patients treated with crizotinib (*n* = 40).

Adverse Events	Grade					
	Any, *n* (%)	1, *n* (%)	2, *n* (%)	3, *n* (%)	4, *n* (%)	5, *n* (%)
Any	36 (90.0)	30 (75.0)	15 (37.5)	13 (32.5)	0	1 (2.5)
In ≥10% of patients						
Elevated ALT	15 (37.5)	11 (27.5)	1 (2.5)	3 (7.5)	-	-
Elevated AST	13 (32.5)	10 (25.0)	2 (5.0)	1 (2.5)	-	-
Nausea	9 (22.5)	7 (17.5)	2 (5.0)	-	-	-
Diarrhea	8 (20.0)	6 (15.0)	2 (5.0)	-	-	-
Elevated ALP	7 (17.5)	3 (7.5)	2 (5.0)	2 (5.0)	-	-
Paresthesia	7 (17.5)	3 (7.5)	4 (10.0)	-	-	-
Constipation	6 (15.0)	5 (12.5)	1 (2.5)	-	-	-
Skin rash	5 (12.5)	1 (2.5)	4 (10.0)	-	-	-
Edema	5 (12.5)	3 (7.5)	2 (5.0)	-	-	-
Visual disturbance	5 (12.5)	5 (12.5)	-	-	-	-
Vomiting	4 (10.0)	3 (7.5)	1 (2.5)	-	-	-
Asthenia/fatigue	4 (10.0)	2 (5.0)	1 (2.5)	1 (2.5)	-	-

ALP, alkaline phosphatase; ALT, alanine aminotransferase; AST, aspartate aminotransferase.

## Data Availability

The data presented in this study are available on request from the corresponding author. The data are not publicly available due to institutional data-sharing restrictions.

## Supplementary Materials

The following supporting information can be downloaded at: https://www.mdpi.com/article/10.3390/cancers16030528/s1, Figure S1: Kaplan–Meier curves of progression-free survival in patients with and without brain metastasis; Table S1. Treatment pattern of crizotinib. Table S2. Cell-free DNA results of patients with ROS1-rearranged NSCLC.
