# Supplementary material for: Real-World Outcomes of Crizotinib in ROS1-Rearranged Advanced Non-Small-Cell Lung Cancer
Source: Cancers (Basel). 2024 Jan 26;16(3):528. doi: 10.3390/cancers16030528 (PMC10854608; doi:10.3390/cancers16030528)

Supplementary Information

Table S1. Treatment pattern of crizotinib

**Characteristics**	**Total (n = 40)**
**Patients with dose adjustments**	15 (37.5)
Adverse events	14 (35.0)
Investigator decision	1 (2.5)
**Last adjusted dose of crizotinib**	
250 mg twice daily	4 (10.0)
250 mg once daily	1 (2.5)
200 mg twice daily	5 (12.5)
200 mg once daily	1 (2.5)
100 mg twice daily	1 (2.5)
Final discontinuation	3 (7.5)
**Discontinued treatment**	21 (50.0)
Disease progression[a]	14 (35.0)
Adverse event[a]	3 (7.5)
Withdrawal of consent	1 (2.5)
Patient refusal	1 (2.5)
Death	2 (5.0)
**Ongoing treatment at data cutoff**	19 (47.5)

[a] One patient had both toxicity and disease progression, leading to the final discontinuation of crizotinib.

Table S2. Cell-free DNA results of patients with ROS1-rearranged NSCLC

**Characteristics**	**No. (%)**
**At the time of crizotinib initiation (n = 14)**	
CD74-ROS1 fusion	2 (14.3)
**At the time of disease progression (n = 8)**	
ROS1 p.(G2032R) c.6094G>A	1 (12.5)
ROS1 p.(D2033N) c.6097G>A	1 (12.5)
EML4-ALK fusion	1 (12.5)
KRAS p.(G12D) c.35G>A	1 (12.5)
KIF5B-RET fusion	1 (12.5)

Figure S1. Kaplan–Meier curves of progression-free survival in patients with and without brain metastasis.

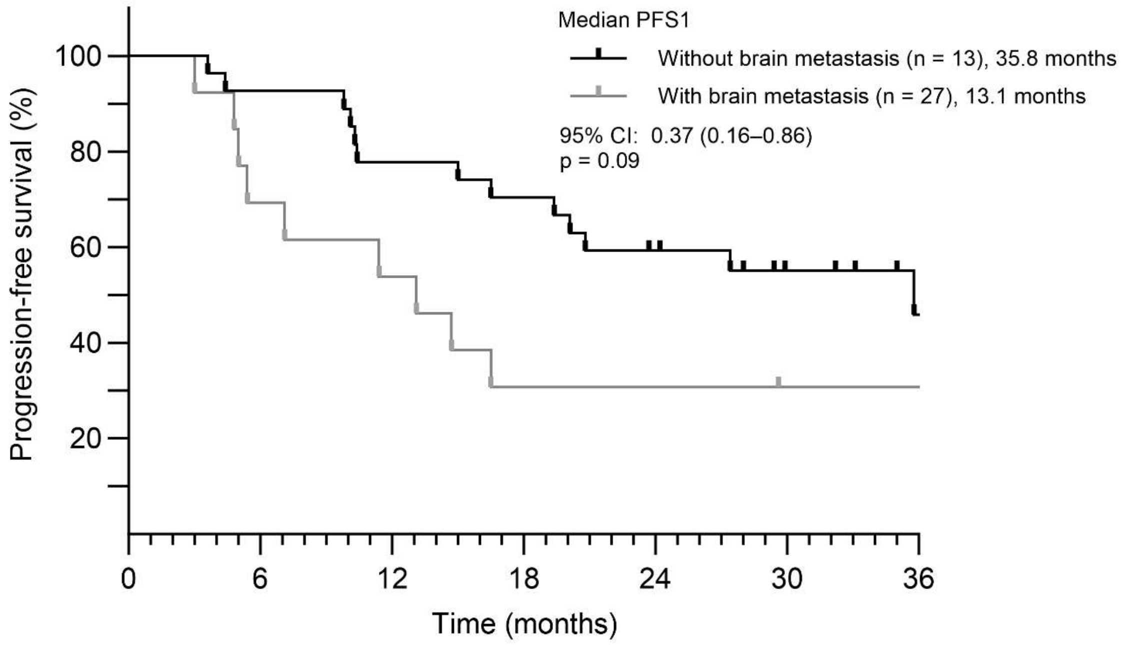

Supplement: Supplementary file 1 [file cancers-16-00528-s001.zip › cancers-2828459-supplementary.pdf]
